# Supplementary material for: Trial-wise awareness ratings do not capture the dynamics of masked priming
Source: Neurosci Conscious. 2026 Jul 6;2026(1):niag034. doi: 10.1093/nc/niag034 (PMC13336397; doi:10.1093/nc/niag034)
Supplement: Supplementary_materials_niag034 [file supplementary_materials_niag034.pdf]

Supplementary Material

Trial-wise awareness ratings do not capture the dynamics of masked priming

Alexander Berger[1,2]*, Michaela Rohr[3], Dirk Wentura[3], & Markus Kiefer[1,2]

[1]Department of Psychiatry, Ulm University, Ulm, Germany

[2]Neuroscience Center Ulm, Ulm University, Ulm, Germany

[3]Department of Psychology, Saarland University, Saarbrücken, Germany

A: Determining window sizes of moving averages for effect course analyses 2

B: Wentura, Rohr, & Kiefer (2025) – Experiment 1a 5

C: Wentura, Rohr, & Kiefer (2025) – Experiment 1b 8

D: Wentura, Rohr, & Kiefer (2025) – Experiment 2: Sample with PAS-ratings depending on distribution of prime conditions 11

E: Influence of the window size and of the threshold for the cluster forming algorithm on the effect course analysis' outcome 13

F: Changes in PAS-ratings separated by PAS-levels 14

Supplementary references 16

A: Determining window sizes of moving averages for effect course analyses

The present work investigated in three different experiments how subjective awareness ratings (PAS) and corresponding priming effects evolve with practice using effect course analysis. These experiments differ with regard to the number of experimental conditions, trials and subjects. As effect course analysis relies on smoothing of data using moving averages to reduce the influence of single-trial noise, we chose a data-driven approach to determine window sizes of moving averages for effect course analysis in the different experiments.

Appropriate window sizes should balance the reduction of single-trial noise with the ability to detect short-term changes in effects (Berger, Kunde, & Kiefer, 2024), thus, the window size should be as small as possible to not conceal any effect changes while simultaneously removing noise. For a visualization of the impact of the window size onto the evolvement of PAS-ratings in Experiment 1 of Wentura and colleagues (2025), see *Figure S1*.

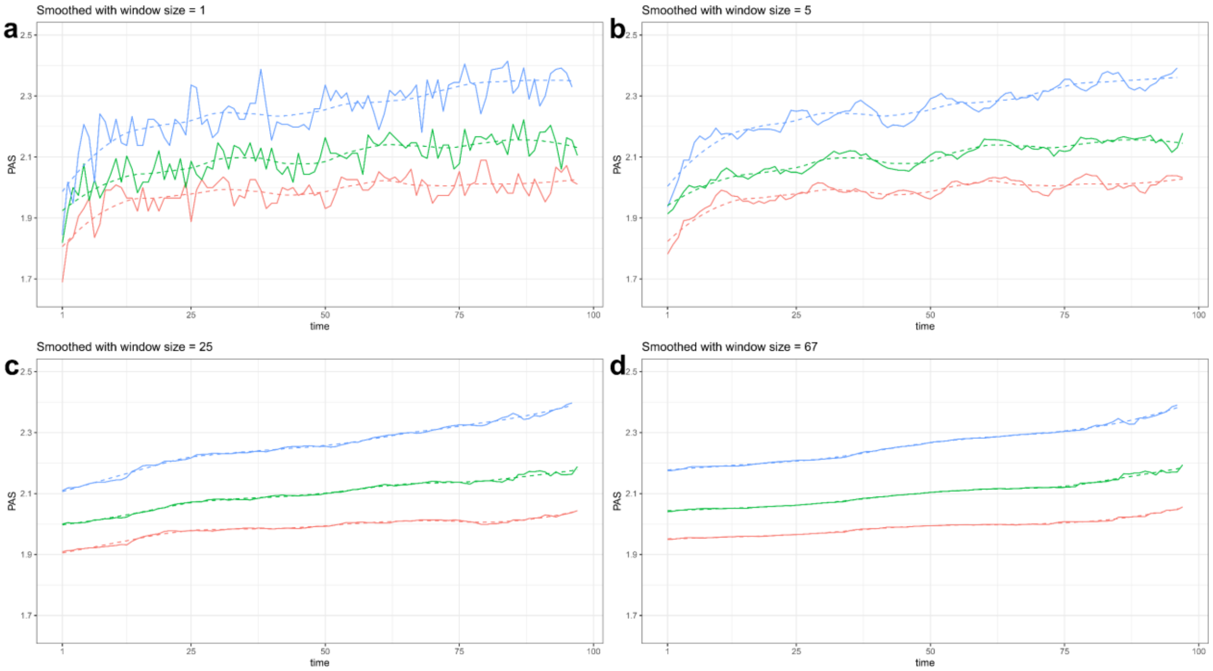

Figure S1 PAS-ratings across trials (time) in Experiment 1 of Wentura, Rohr, & Kiefer (2025) for the different prime durations smoothed with different window sizes of moving averages. Depicted are the smoothed data (solid lines) and a fitted line (dashed lines, calculated per loess fitting in R). Window sizes for smoothing were 1 trial (Panel a, i.e., no smoothing), 5 trials (Panel b), 25 trials (Panel c), and 67 trials (Panel d, minimum of available trials per subject and condition, i.e., maximal possible number of trials for smoothing).

As is evident in *Figure S1*, panels a and b, the development of PAS-ratings is best described by a steep initial increase, followed by a flatter increase. However, without smoothing (Panel a), also large single-trial fluctuations are present (although the data is averaged across N = 116 subjects). This noise is gradually reduced with larger window sizes (panels b – d). However, too extreme smoothing (e.g., panels c and d) also alters the general course of PAS-ratings, for instance concealing the sharp initital increase. An appropriate window size should retain the general trend in the data (dashed lines in panels a and b), but reduce noise ("fluctuations" in solid lines around the dashed lines). To achieve these goals, the following algorithm was used to determine appropriate window sizes for the different effects tested in the present study:

1) The data was smoothed using moving averages with a small window size. Here, we used a window size of 5 trials, which should not conceal more global trends, but which should reduce large single-trial fluctuations which might impair fitting a trend line (see step 2).
2) This slightly smoothed data was fitted with a "loess" function (as implemented in R; R Core Team, 2020) to obtain the temporal trend underlying the data. The span of the loess function was set to 0.3 – 0.8 (determined by visually inspecting whether the general temporal trend was captured by the chosen span).
3) The data was smoothed with different window sizes. For each window size, the standard deviation (SD) of the difference between the smoothed data and the fitted line obtained from step 2 was calculated as: $SD_{diff} = SD(data_{fitted,t} - data_{smoothed\ with\ ws,t})$, with *t* indicating the time point/trial, and *ws* indicating the tested window size.
4) Step 3 calculates the SD of the deviation of smoothed data from the fitted line, with larger absolute deviations from the fitted line resulting in a larger SD. Thus, the more noise remaining in the smoothed data, the larger SD_{diff}. Furthermore, the more the smoothed data deviates from the general trend, the larger this SD as well. The optimal window size was determined as the chosen setting which minimizes SD_{diff}.
5) Steps 1 – 4 were repeated for each condition. The window size was determined as the up-rounded average of all conditions relevant for this comparison (e.g., for all prime durations). If this resulted in an even number, one was added (as window sizes must be odd to achieve a symmetric averaging around the chosen trial).

For the example of PAS-ratings in Experiment 1 of Wentura and colleagues (2025), see *Figure S2*.

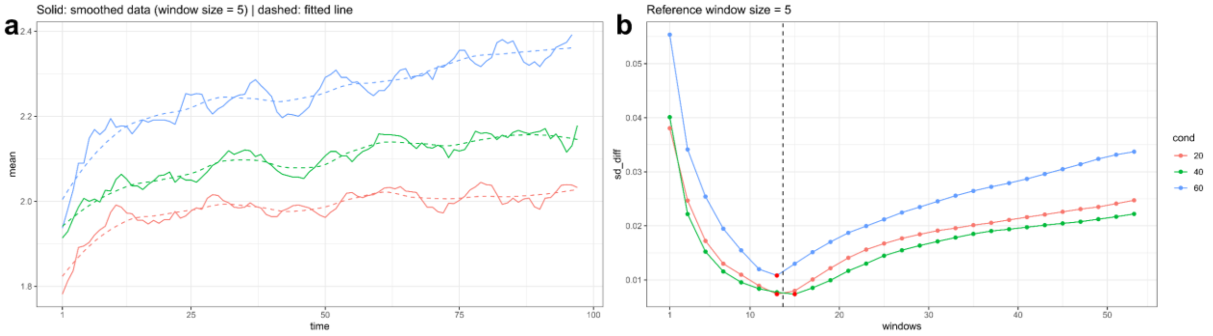

Figure S2 Panel a shows PAS-ratings for the different prime durations in Experiment 1 of Wentura, Rohr, & Kiefer (2025) smoothed with moving averages with a window size of 5. The general trend underlying the data is shown as dashed lines. Panel b shows the SD of the deviation of data smoothed with different window sizes from this general trend. The minimum of this SD_{diff} averaged across all three prime durations is a window size of 13.7 indicated by the vertical dashed line, which transfers to a chosen window size of 15.

This approach was applied to each effect course analysis calculated in the present work. Information regarding the chosen window sizes for Experiment 1 and 2 of Wentura and colleagues (2025) as well as for the data of Kiefer and colleagues (2023) can be found in *Table S1* and *S2*, respectively.

Table S1: Chosen window sizes for the data of Wentura, Rohr, & Kiefer (2025)

Experiment	Dependent variable	Conditions	min(SD_diff)	Chosen window size
1	PAS-ratings	SOA [20ms,40ms,60ms]	[13,15,13]	15
	RTs (sample with PAS-ratings)	Congruency [congruent,incongruent]xSOA[20,40,60]	[9,11,11,7,9,11]	11
	RTs (sample without PAS-ratings)	Congruency [congruent,incongruent]xSOA[20,40,60]	[13,11,13,15,13,7]	13
2	PAS-ratings	SOA [40ms]	[25]	25
	RTs (sample with PAS-ratings)	Congruency [congruent,incongruent]xSOA[40]	[19,15]	17
	RTs (sample without PAS-ratings)	Congruency [congruent,incongruent]xSOA[40]	[29,25]	27
1a	PAS-ratings	SOA [20ms,40ms,60ms]	[17,19,25]	21
	RTs (sample with PAS-ratings)	Congruency [congruent,incongruent]xSOA[20,40,60]	[13,13,13,11,11,15]	13
	RTs (sample without PAS-ratings)	Congruency [congruent,incongruent]xSOA[20,40,60]	[13,17,13,11,13,11]	13
1b	PAS-ratings	SOA [20ms,40ms,60ms]	[13,13,13]	13
	RTs (sample with PAS-ratings)	Congruency [congruent,incongruent]xSOA[20,40,60]	[9,11,9,9,9,7]	9
	RTs (sample without PAS-ratings)	Congruency [congruent,incongruent]xSOA[20,40,60]	[11,11,11,11,13,13]	13

Note: Min(SD_diff) indicates the window size of each condition, for which SD_diff was minimized. The chosen window size is the (up-rounded) average of these values (plus 1, if this is an even number).

Table S2: Chosen window sizes for the data of Kiefer, Harpaintner, Rohr, & Wentura (2023)

Dependent variable	Conditions	min(SD_diff)	Chosen window size
PAS-ratings	SOA [20ms,40ms,60ms]	[29,15,29]	25
RTs (sample with PAS-ratings)	Semantic relatedness [related,unrelated]xSOA[20,40,60]	[15,15,13,15,15,11]	15
RTs (sample without PAS-ratings)	Semantic relatedness [related,unrelated]xSOA[20,40,60]	[13,15,15,17,17,15]	17

Note: Min(SD_diff) indicates the window size of each condition, for which SD_diff was minimized. The chosen window size is the (up-rounded) average of these values (plus 1, if this is an even number).

B: Wentura, Rohr, & Kiefer (2025) – Experiment 1a

Sample with PAS-ratings

Considering trial-wise awareness ratings, effect course analysis revealed one significant cluster, where PAS-ratings differed as a function of prime duration: $F = 321.9$, $p = .002$, trials 39 – 98. PAS-ratings for the three different prime durations increased with practice, while significant differences between prime durations were only observed in the second half of the experiment. PAS-ratings for catch trials increased similarly compared to trials where a prime stimulus was actually presented, see *Figure S3*, Panel a.

Regarding the corresponding priming effects, no cluster was observed for the 20ms prime duration at all. For the 40ms condition, two significant clusters were observed, $T = 22.6$, $p = .037$, trials 1 – 11, and $T = 37.1$, $p = .006$, trials 13 – 27. Priming was significant at the beginning of the experiment, but vanished with further practice. Similarly, priming was maximal at the beginning and vanished with practice also for the 60ms prime duration. However, here, the corresponding cluster did not reach significance, $T = 17.9$, $p = .065$, trials 1 – 8, see *Figure S3*, Panel b.

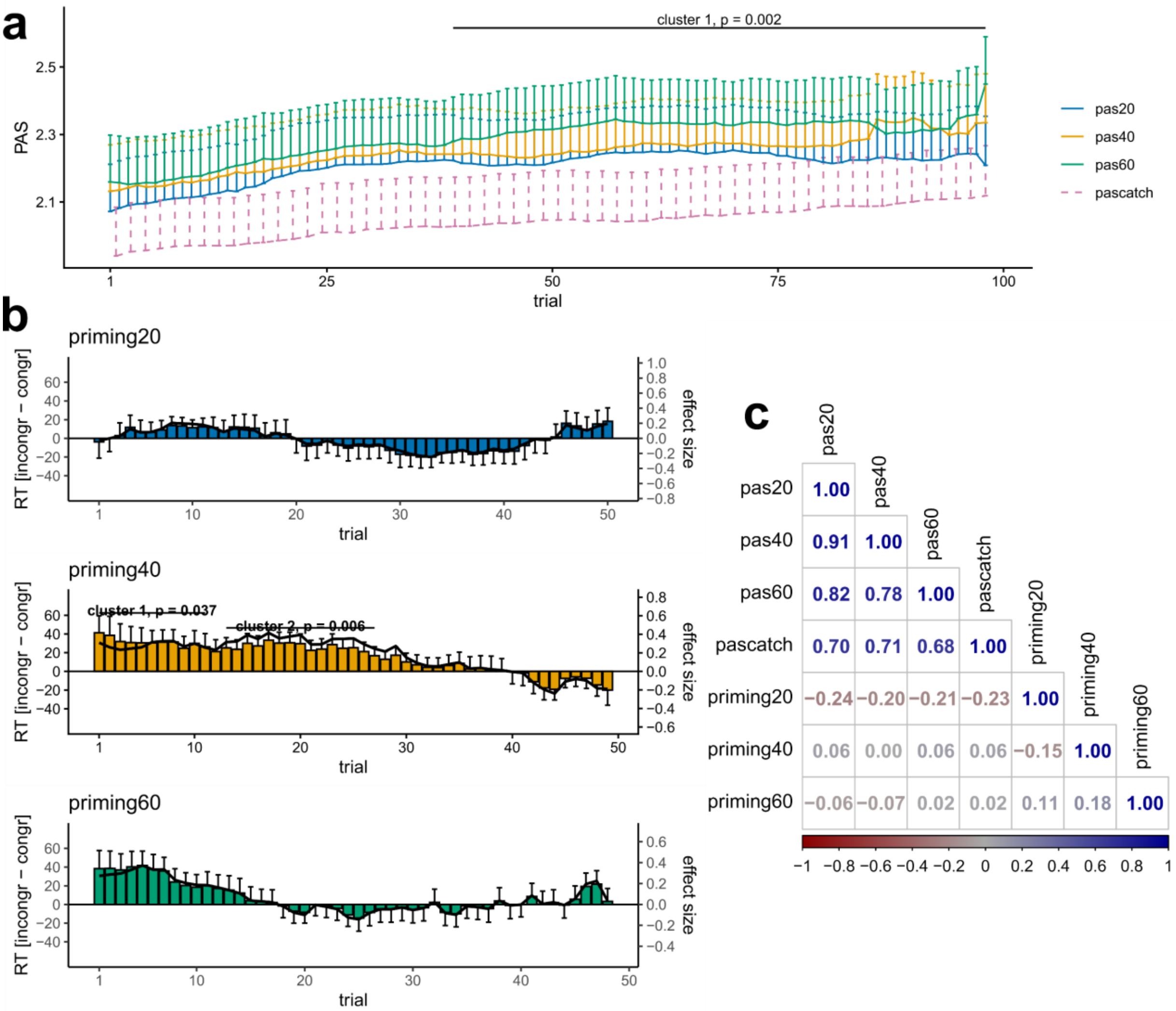

Figure S3 Results for the sample with PAS-ratings in Experiment 1a of Wentura, Rohr, & Kiefer (2025). Panel a shows the development of PAS-ratings for the different prime durations (20, 40, and 60ms) as well as catch trials, with all PAS-ratings increasing with practice. Panel b shows effect courses for the RT response priming effect for the different prime durations. For the 40 and 60ms prime duration, priming was maximal at the beginning of the

experiment (however only reflected by significant clusters for the 40ms prime duration) but vanished with further practice. Panel c shows the correlations of individual slopes extracted from the analyses in panels a and b. Slopes of priming effects did not correlate with each other, nor with slopes of PAS-ratings. However, slopes of PAS-ratings, including those of catch trials, substantially correlated with each other.

Hence, corroborating the analysis reported in the main text, PAS-ratings (even for catch trials) increased with practice, while corresponding priming effects showed a different time course, i.e., decreasing with practice. This was also reflected in the correlation analysis (*Figure S3*, Panel c), where between all individual slopes of PAS-ratings (including catch trials) substantial correlations were observed, all rs > .68, all ps_{Holm} < .001. In contrast, no significant correlations were observed between slopes of priming effects with each other nor between priming effects and PAS-ratings, all $|r|$s < .25, all ps_{Holm} > .999. A repeated-measures ANOVA (rm-ANOVA) with slopes of PAS-ratings as dependent variable showed no significant difference between prime durations and catch trials, $F(3,156) = 1.29$, $p = .281$. Subsequent contrasts showed the increase in PAS-ratings for catch trials to be significant ($p = .022$). However, PAS-ratings in the conditions with a prime showed no signfiicant more pronounced increase compared to that of catch trials, for all prime durations (all ps > .246). For slopes of priming scores, a corresponding rm-ANOVA showed again no significant differences between prime durations, $F(2,104) = 2.04$, $p = .136$, and subsequent contrasts showed no significant difference from zero for these slopes for the 20ms prime duration ($p = .797$). The slopes for the 40ms ($p = .055$) and 60ms condition ($p = .150$) were more negative than that for the 20ms prime duration, with this difference however not reaching significance.

Sample without PAS-ratings

In the sample without PAS-ratings, effect course analysis revealed no significant cluster in the 20ms prime duration (all ps > .160). For the 40ms prime duration, no significant priming was observed as well. However, one cluster at the end of the experiment showed positive priming, however lacking significance, $T = 14.3$, $p = .097$, trials 37 – 41. Regarding the 60ms prime duration, a cluster at the experiment's beginning failed to reach significance, $T = 16.5$, $p = .080$, trials 1 – 7, while significant priming was observed at the end of the experiment, $T = 50.0$, $p = .002$, trials 30 – 47, see *Figure S4*, Panel a. Moreover, the slopes of priming scores did not correlate significantly with each other (all $|r|$s < .16, all ps_{Holm} > .868; *Figure S4*, Panel b). A subsequent rm-ANOVA showed no significant effect of prime duration on these slopes, $F(2,102) = 0.19$, $p = .832$. Follow-up contrasts confirmed that slopes of priming scores did neither deviate significantly from zero in the 20ms condition ($p = .155$), nor did the slopes in the 40ms ($p = .721$) and 60ms ($p = .547$) prime duration differ significantly from those of the 20ms condition.

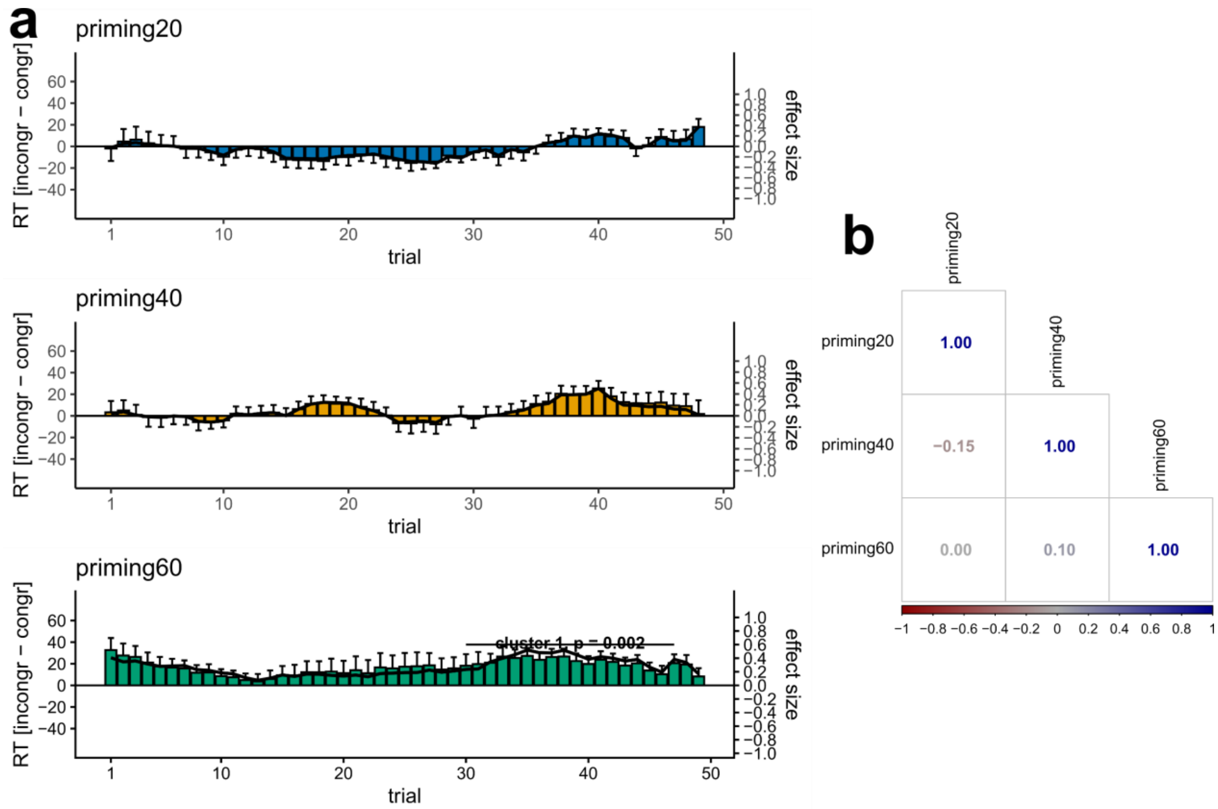

Figure S4 Results for the sample without PAS-ratings in Experiment 1a of Wentura, Rohr, & Kiefer (2025). Panel a shows the effect courses for the priming effects, separately per prime duration. For the 60m prime duration, (positive) priming emerged, which was most reflected by a significant cluster at the end of the experiment. For the other prime durations, no significant priming was observed. Panel b shows correlations between slopes of individual priming scores per participant, though no significant correlations were observed.

C: Wentura, Rohr, & Kiefer (2025) – Experiment 1b

Sample with PAS-ratings

In Experiment 1b, effect course analysis of PAS-ratings revealed one significant cluster, spanning across the whole experimental duration, $F = 3473.0$, $p < .001$, trials 1 – 93. In contrast to Experiment 1a (compare *Figure S3*, Panel a), differences between the prime durations were larger (*Figure S5*, Panel a). Moreover, PAS-ratings (including catch trials) increased with practice, with this increase however being less pronounced compared to Experiment 1a.

Effect course analysis of the priming effects revealed no cluster at all for the 20 and 40ms prime duration. For the 60ms condition, no significant cluster was observed as well (all *p*s > .165), see *Figure S5*, Panel b.

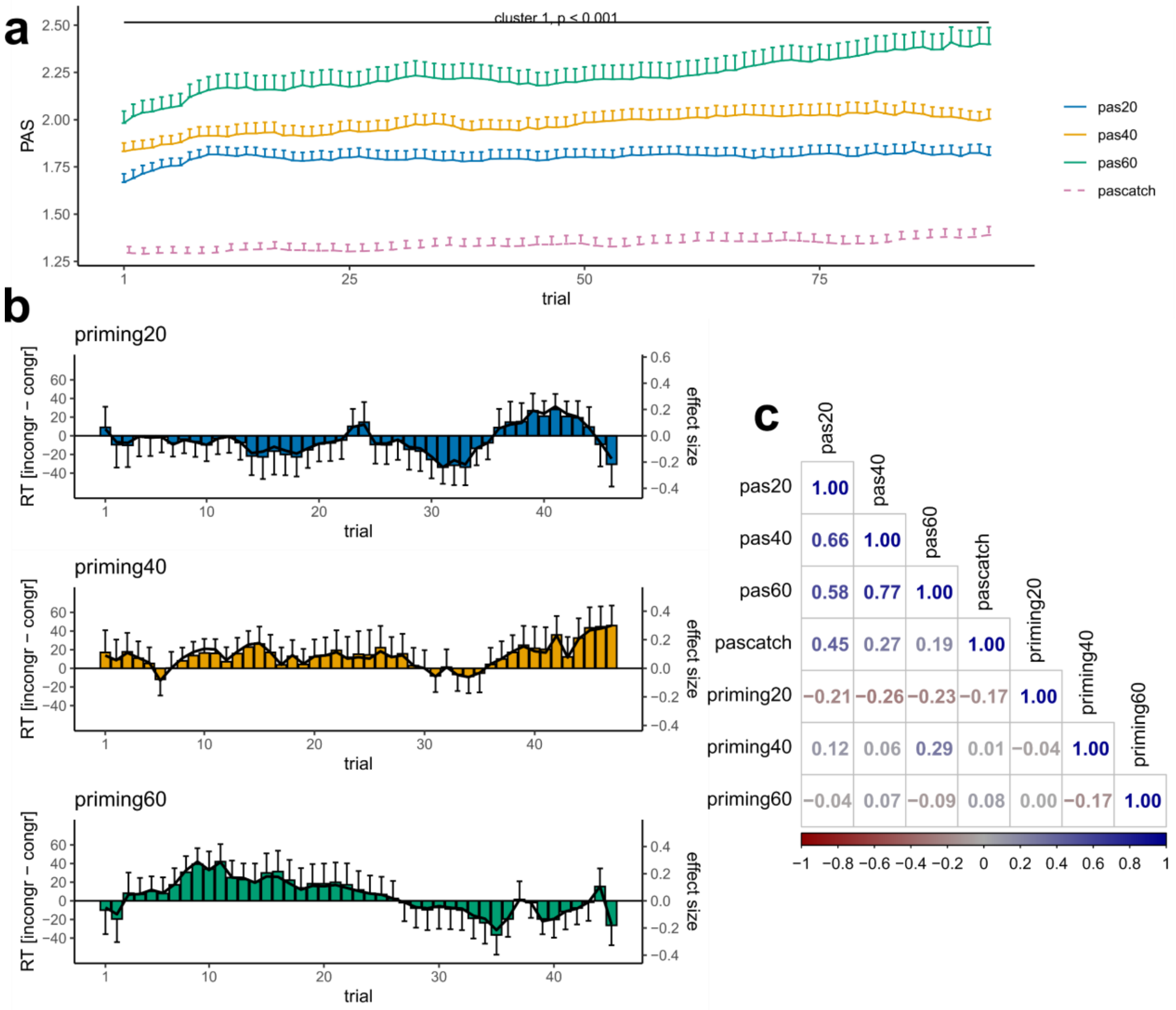

Figure S5 Results for the sample with PAS-ratings in Experiment 1b of Wentura, Rohr, & Kiefer (2025). Panel a shows the development of PAS-ratings for the different prime durations (20, 40, and 60ms) as well as catch trials. Panel b shows effect courses for the RT response priming effect for the different prime durations. No significant priming was observed. Panel c shows the correlations of individual slopes extracted from the analyses in panels a and b. Only between slopes of PAS-ratings, robust correlations were observed (despite of PAS-ratings for catch trials, which correlated only significantly with PAS-ratings for the 20ms prime duration).

Thus, like for Experiment 1a, PAS-ratings showed an increasing trend, while for the corresponding priming effects no such trend could be observed. As a consequence, correlation

analysis (*Figure S5*, Panel c) showed substantial correlations only between PAS-ratings for the three different prime durations, all *r*s > .57, all $ps_{\text{Holm}} < .001$. However, in contrast to Experiment 1a, the correlations between slopes of PAS-ratings for catch trials with those for the different prime durations were less pronounced, and reached only significance for the correlation with PAS-ratings of the 20ms prime duration, $r = .45$, $p_{\text{Holm}} = .004$, both other *r*s < .28, both $ps_{\text{Holm}} > .473$. Likewise, between slopes of priming scores, as well as slopes of priming scores and those of PAS-ratings, no significant correlations emerged, all |*r*|s < .30, all $ps_{\text{Holm}} > .345$. The rm-ANOVA showed individual slopes of PAS-ratings to significantly differ across prime durations including catch trials, $F(3,186) = 5.40$, $p = .001$. While contrasts revealed slopes in catch trials to not significantly differ from zero ($p = .095$), the increase in the 60ms prime duration was significant larger compared to that of catch trials ($p = .002$). The slopes of PAS-ratings in the 20ms and 40ms condition, however, did not differ significantly from catch trials (both *p*s > .448). Regarding the corresponding analysis of slopes of priming scores, the rm-ANOVA did not reveal a significant effect of prime duration, $F(2,124) = 1.57$, $p = .211$. Likewise, follow-up contrasts showed no deviation of these slopes from zero for the 20ms prime duration ($p = .827$). Furthermore, neither the slopes in the 40ms nor 60ms condition differed significantly from those of the reference category (20ms; both *p*s > .172).

Sample without PAS-ratings

In the sample without PAS-ratings in Experiment 1b, no cluster at all was observed in the 20ms prime duration. The 40ms prime duration revealed two clusters with negative priming, with the first one reaching significance, $T = -31.7$, $p = .016$, trials 1 – 12, second cluster: $T = -12.8$, $p = .101$, trials 37 – 42. For the 60ms prime duration, albeit positive priming was observed on a descriptive level (see *Figure S6*, Panel a), this was not reflected by a significant cluster (all *p*s > .209).

The correlation analysis revealed no significant correlations between slopes of priming scores (all |*r*|s < .26, all $ps_{\text{Holm}} > .147$). A rm-ANOVA showed no significant effect of prime duration on slopes of priming scores, $F(2,124) = 0.13$, $p = .880$. Subsequent contrasts revealed non-significant slopes in the 20ms condition ($p = .584$), and slopes in the 40ms and 60ms condition did not differ significantly from those of the 20ms prime duration (both *p*s > .696).

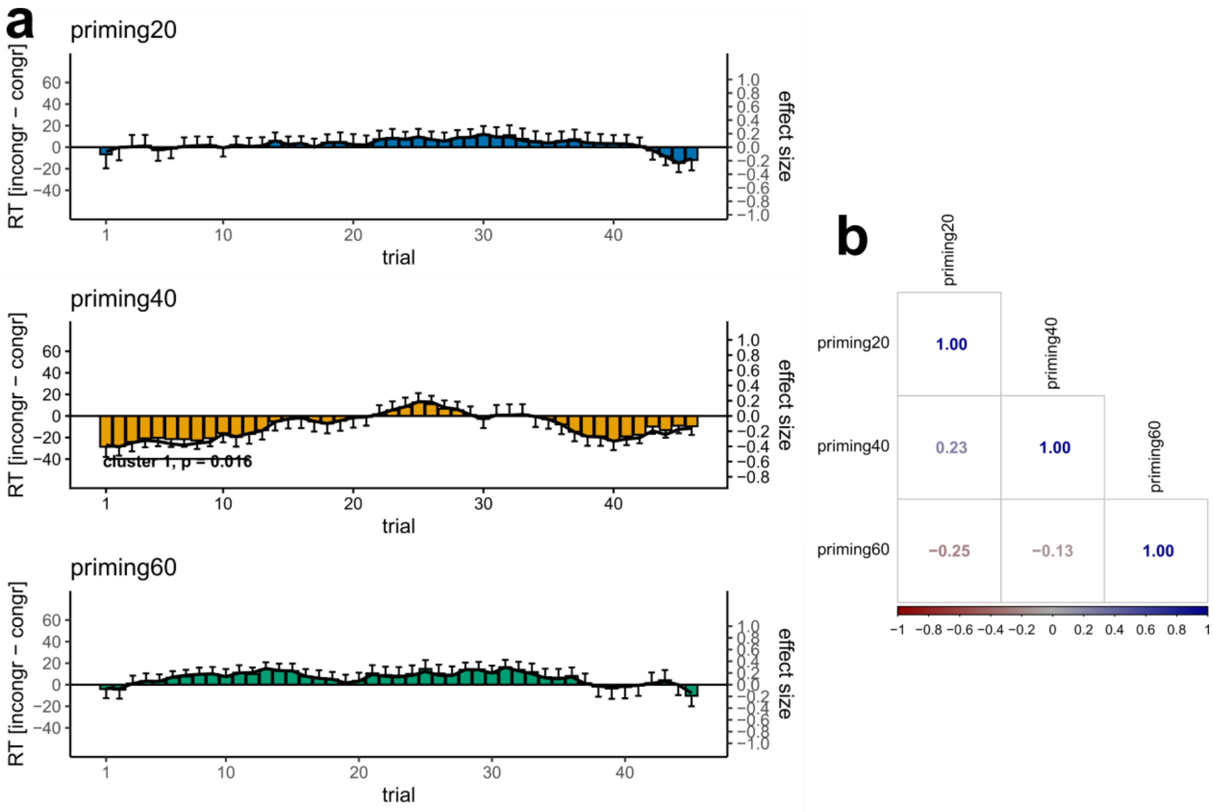

Figure S6 Results for the sample without PAS-ratings in Experiment 1b of Wentura, Rohr, & Kiefer (2025). Only for the 40ms prime duration, significant priming was observed at the beginning of the experiment, which however showed a negative sign, indicating faster responses for the incongruent condition (panel a). Correlations analysis of individual slopes extracted from panel a showed no significant correlations.

D: Wentura, Rohr, & Kiefer (2025) – Experiment 2: Sample with PAS-ratings depending on distribution of prime conditions

Due to an error in the experimental program in Experiment 2 of Wentura and colleagues (2025), in the sample with PAS-ratings only for around half of the participants the distribution of conditions was balanced. In detail, for 60 participants, trials were equally distributed across the 2 (prime: sad vs. anger) × 2 (target: sad vs. anger) design, i.e., 90 trials per condition. For the remaining 62 participants this distribution was not fully balanced. There, the number of trials in each cell ranged from 60 – 120, with the total number of all trial types always being 360. However, on the group level, congruent and incongruent trials were here balanced as well.

There were no differences in average priming effects between these two samples, as documented in a correction submitted to the journal of the original article. Nevertheless, we re-ran effect course analyses in the sample with PAS-ratings of Experiment 2 separately per groups with an equal and unequal distribution of prime-target conditions, to assess whether this error caused any chances in the dynamics of PAS-ratings or priming effects. As can be seen below, there were no differences in the pattern of effects between both groups.

Equal distribution

Effect course analysis of the priming effect revealed no significant cluster. Furthermore, correlation analysis showed only a significant correlation between slopes of PAS-ratings for trials with a prime and catch trials (r = .57, p_{Holm} < .001), but not between slopes of priming scores with any slope of PAS-ratings (both $|r|$s < .08, both ps_{Holm} > .999). A subsequent rm-ANOVA revealed slopes of PAS-ratings to not differ between catch trials and trials with a prime, $F(1,59)$ = 1.85, p = .179. Nevertheless, follow-up contrasts showed the mean increase in PAS-ratings for catch trials to be significant (p = .039), while slopes in the 40ms prime duration did not differ significantly from that of catch trials (p = .179). Moreover, slopes of priming scores did not differ significantly from zero (p = .905).

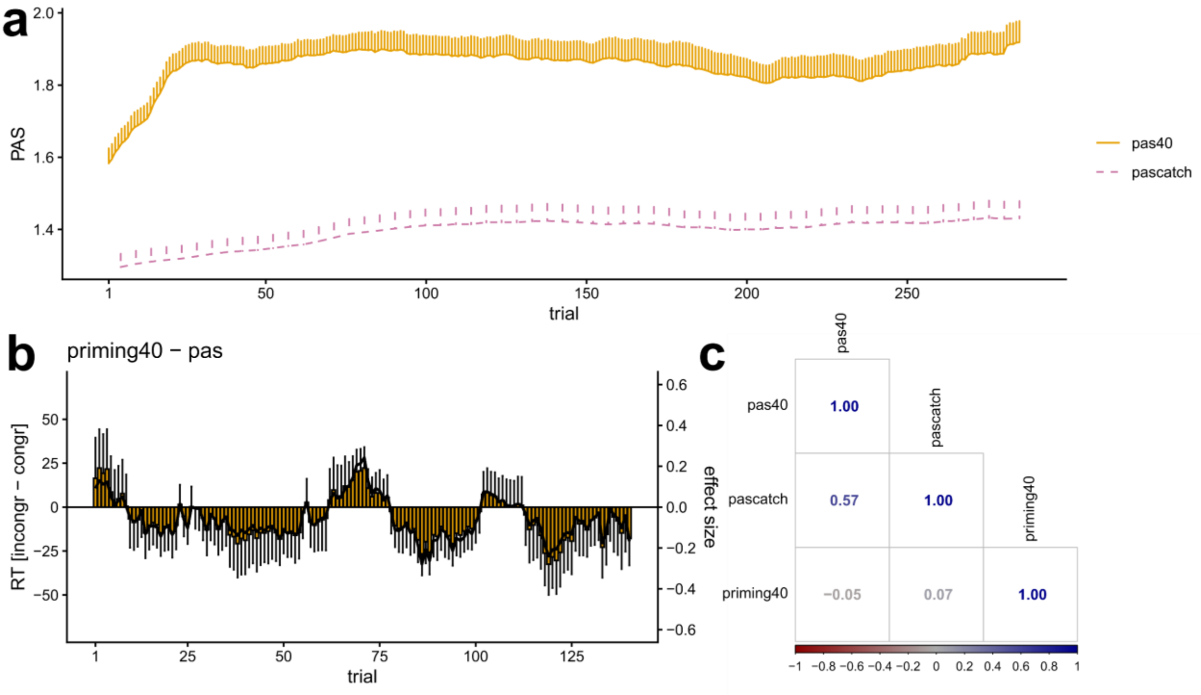

Figure S7 Results for participants receiving a fully balanced distribution of prime-target pair conditions among the sample with PAS-ratings in Experiment 2 of Wentura, Rohr, & Kiefer (2025). No significant priming emerged, and correlation analysis of individual slopes showed only a significant correlation between PAS-ratings for catch trials and the 40ms prime duration (r = .57, p_{Holm} < .001).

Unequal distribution

Similar to the sample with an equal distribution of prime-target pair conditions, the analysis of the sample with an unequal distribution also paralleled the conjoint analysis of both samples as documented in the main text: Effect course analysis revealed no significant cluster reflecting priming, and correlation analysis revelaed only a significant correlation between slopes of PAS-ratings (r = .60, p_{Holm} < .001), but not between slopes of priming scores with any slope of PAS-ratings (both $|r|$s < .21, both $p\text{s}_{\text{Holm}}$ > .223). The subsequent rm-ANOVA of individual slopes of PAS-ratings revealed a significant difference between trials with a prime and catch trials, $F(1,61) = 4.27$, p = .043. Subsequent contrasts revelaed a significant increase in PAS-scores for catch trials (p = .003). Slopes in the 40ms prime duration showed a significantly less steep increase compared to catch trials (p = .043). Slopes of priming scores did not differ significantly from zero (p = .282).

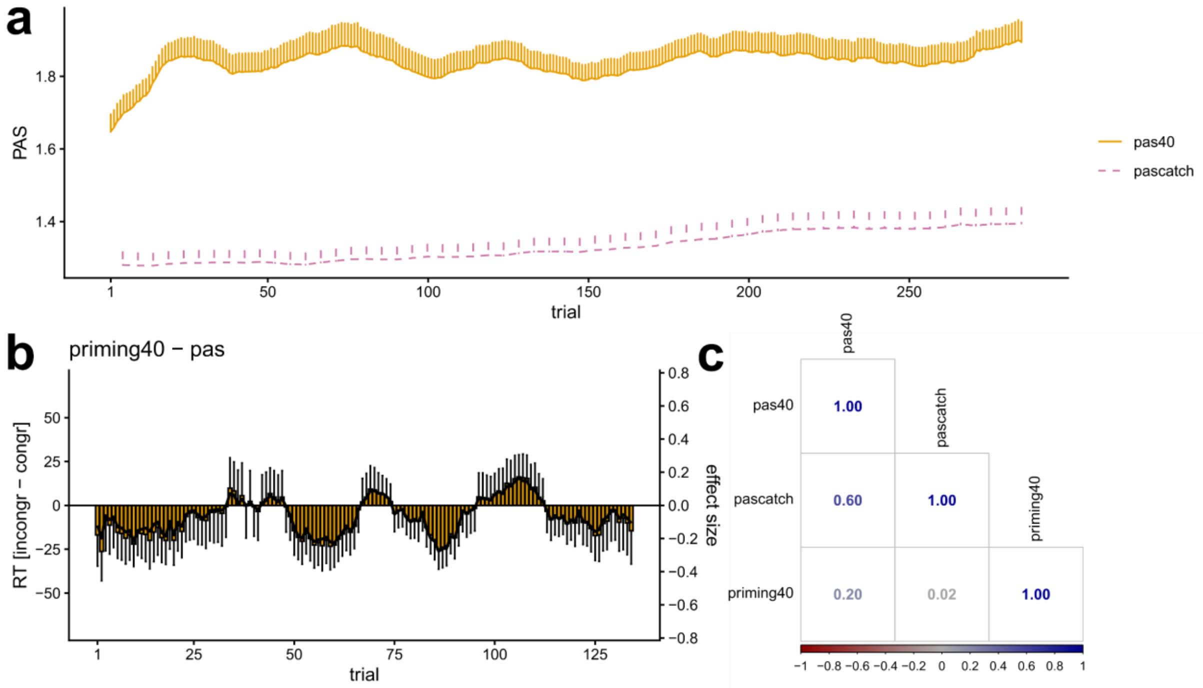

Figure S8 Results for participants receiving an unbalanced distribution of prime-target pair conditions among the sample with PAS-ratings in Experiment 2 of Wentura, Rohr, & Kiefer (2025). No significant priming emerged, and correlation analysis of individual slopes showed only a significant correlation between PAS-ratings for catch trials and the 40ms prime duration (r = .60, p_{Holm} < .001).

E: Influence of the window size and of the threshold for the cluster forming algorithm on the effect course analysis' outcome

To illustrate the influence of the effect course analysis' settings on its outcome, we assessed the impact of the window size for smoothing as well as the threshold *p*-value for grouping below-threshold trials together to clusters on the *p*-value and length of the largest observed cluster (500 permutations). These analyses were exemplarily conducted on the data of the sample with PAS-ratings in Experiment 1 of Wentura et al. (2025), see *Figure S9*.

To summarize, the effect course analysis appears rather consistent in whether it detects a significant cluster or not (see also Supplementary Material J in Berger et al., 2024), as, e.g., reflected by PAS-ratings (consistent presence) and priming in the 20ms condition (consistent absence). For the 40ms and 60ms condition, the presence of a significant cluster depends on a large enough window size paired with a not too conservative threshold, which is not surprising (e.g., if the threshold is too small no t-test at the trial/sample level would fall short of this threshold, which is similar for small window sizes where larger noise is present). For the 40ms prime duration, the chosen settings for the analysis reported in the main text (window size = 11, threshold = 0.1) failed to provide a significant cluster. For larger window sizes as well as more liberal thresholds, a significant cluster was observed. This suggests that more methodological work is needed to identify suitable thresholds for the cluster forming algorithm. Note that generally choosing larger window sizes is no solution for this problem, as with larger window sizes temporal information gets lost, and there is always a tradeoff between temporal resolution and reduction of noise. With this regard, the length of the cluster (number of included trials) increased with larger window sizes.

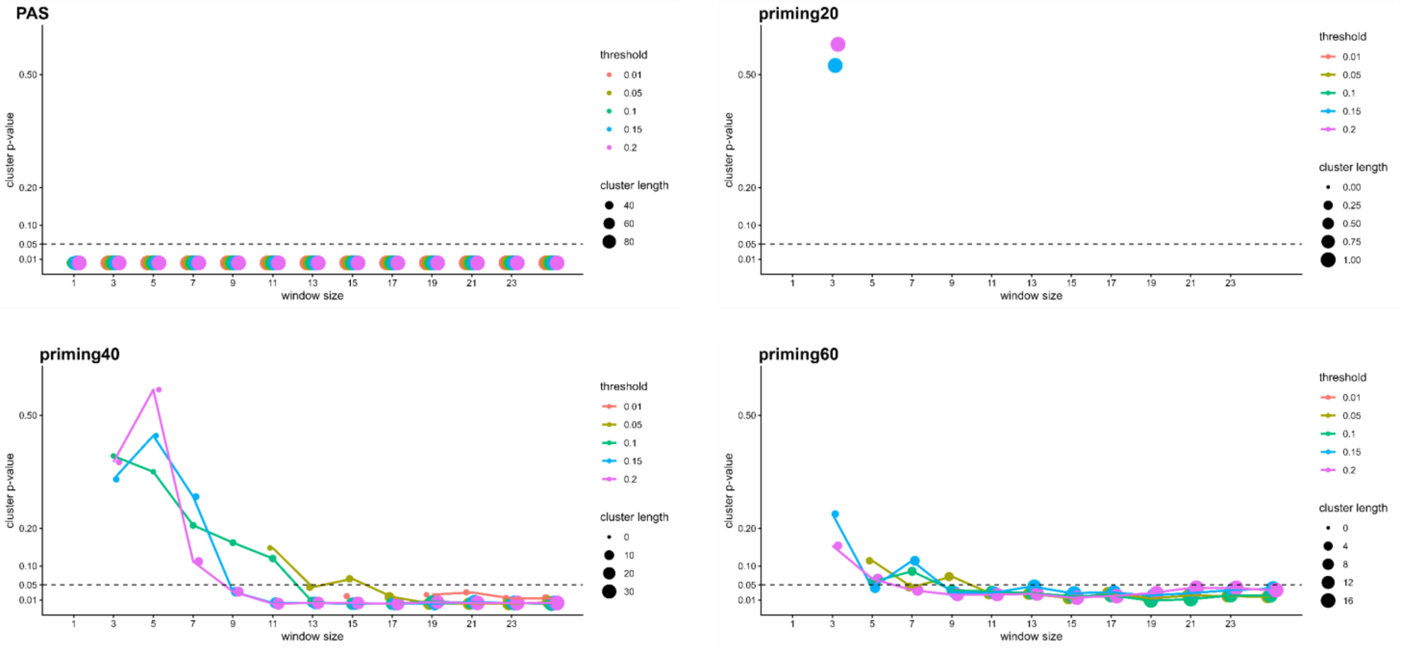

Figure S9 Influence of the chosen window size (x-axis) and the threshold for the cluster forming algorithm (color) on the largest observed cluster in the data of Experiment 1 of Wentura, Rohr, & Kiefer (2025), sample with PAS-ratings (*Figure 1* in the main text). The lack of a point for any combination indicates that no cluster was determined in the first place. Shown are (from top left to bottom right) the influence on the obtained cluster for PAS-ratings, priming for the 20ms prime duration, as well as priming for the 40 and 60ms prime duration. The presence/absence of a significant cluster is rather consistent across settings (given a sufficient large window size). With a more liberal threshold and stronger smoothing (which, however, conceals temporal information), also for the 40ms prime duration a significant cluster is observed, in contrast to the settings chosen in the main text (window size = 11, threshold = 0.1).

F: Changes in PAS-ratings separated by PAS-levels

The analyses of PAS-ratings reported in the main text treated these ratings as a continuous variable, averaging across levels of PAS-ratings. This was necessary to conduct effect course analysis and to estimate individual slopes of PAS-ratings. Nonetheless, the PAS is originally an ordinal scale, reflecting different (subjective) aspects of perceptual clarity/ awareness (Ramsøy & Overgaard, 2004). Therefore, to estimate how the different levels of PAS-ratings developed with practice, we plotted on a descriptive level the proportion of ratings of a given PAS-level (across participants) at a given trial, separately per prime duration and catch trials, see the figures below. Note that like for the figures of PAS-ratings shown in the main text, the trial scale of catch trials was adjusted to match those of the other prime durations due to a lower number of trials presented for catch trials.

As can be seen in *Figure S10*, for the evaluative response priming data, Experiment 1, the observed increase in PAS-ratings appears to be mainly driven by a decrease of PAS-ratings with level 1, and an increase of PAS-ratings with level 2 (especially for catch trials). Ratings reflecting participants reporting seeing (aspects of) the prime (3 and 4) appear more constant across the experiment.

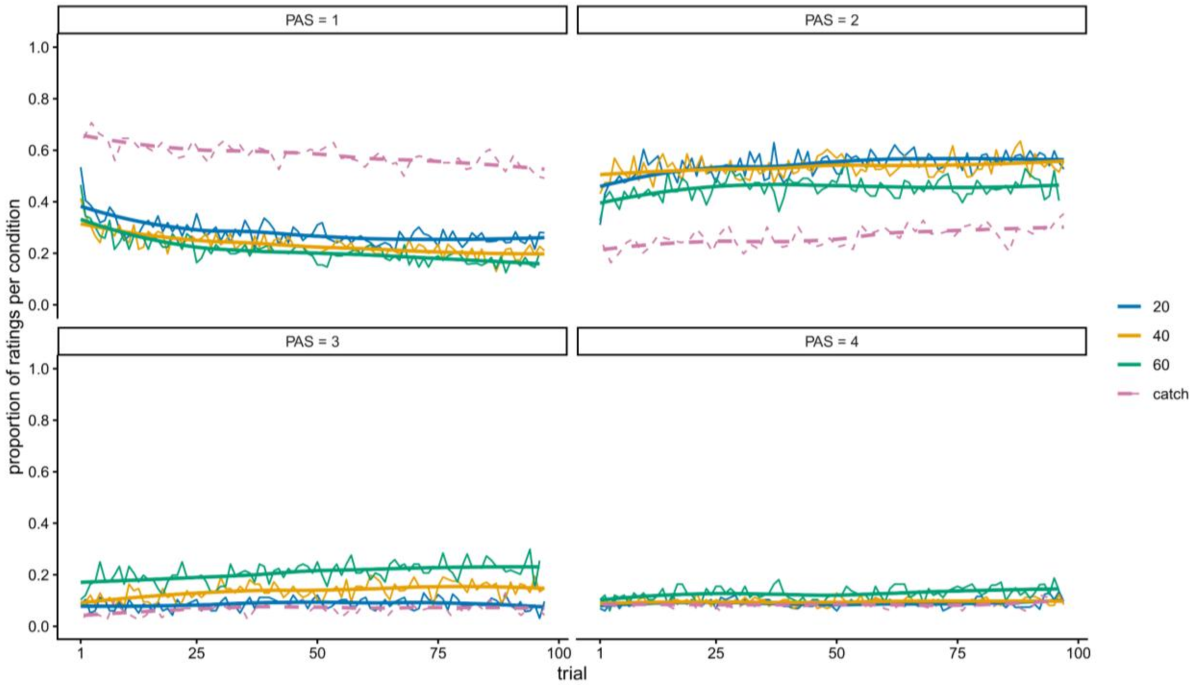

Figure S10 Development of the proportion of ratings per PAS-level in Experiment 1 of Wentura, Rohr, & Kiefer (2025), separately per prime duration and catch trials. Trial-wise proportions were calculated across participants, with the sum of all per-condition PAS-ratings at a given trial set to 1.

Experiment 2 using a fixed prime duration (*Figure S11*) draws a similar picture like Experiment 1. While ratings for PAS-level 1 decreased, level 2 PAS-ratings increased, especially for catch trials. Ratings of level 3 or 4 appear rather constant in contrast. Accordingly, participant became less likely to indicate that they have not seen anything with practice, even for catch trials.

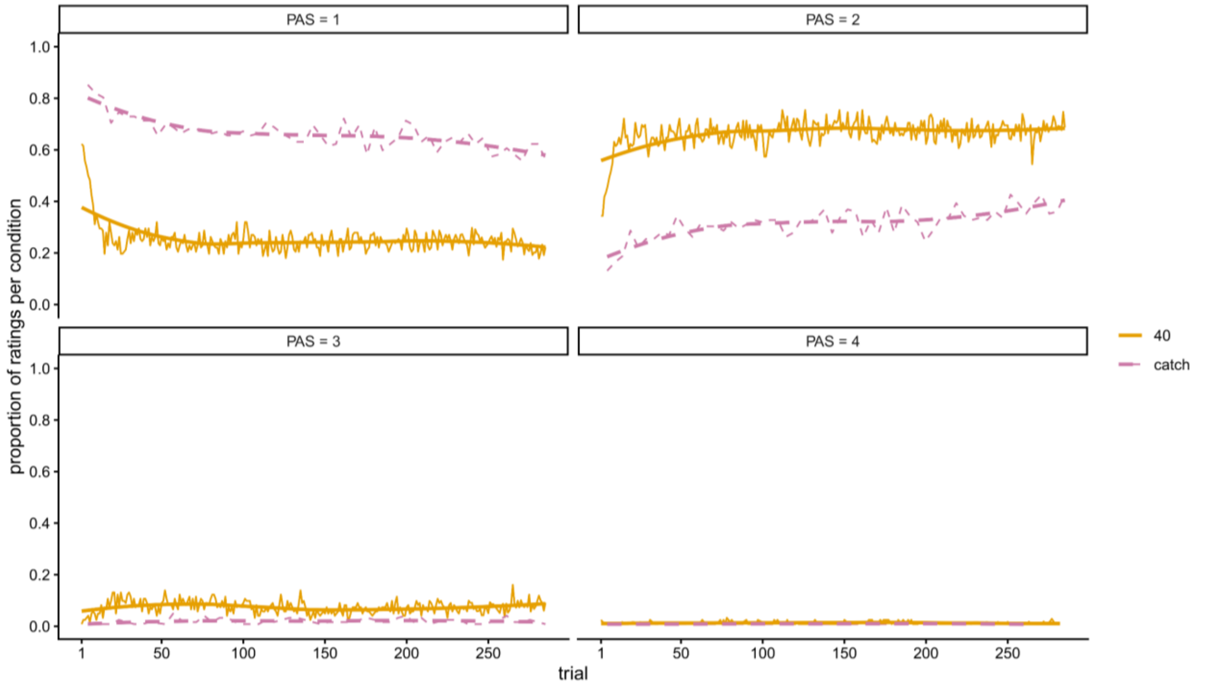

Figure S11 Development of the proportion of PAS-ratings in Experiment 2 of Wentura, Rohr, & Kiefer (2025).

The semantic priming data depicted in *Figure S12* show descriptively for level 1 and 2 PAS-ratings a general decrease in all presentation conditions, while a general increase was observed for level 3 ratings. Level 4 ratings increased mainly for the 60ms presentation condition. As an increase of PAS 3 ratings was also observed for catch trials, this suggests that also in the semantic priming experiment the general increase of PAS-ratings as a function of practice does not exclusively reflect more veridical perceptions during the course of the experiment but also changes in response bias and/or false perceptions. The slightly different time courses of PAS-ratings across priming paradigms indicate that practice-induced changes can vary as a function of the precise experimental paradigm, i.e., stimuli, target task or experiment length (the semantic priming experiment comprised less trials than the evaluative priming experiments).

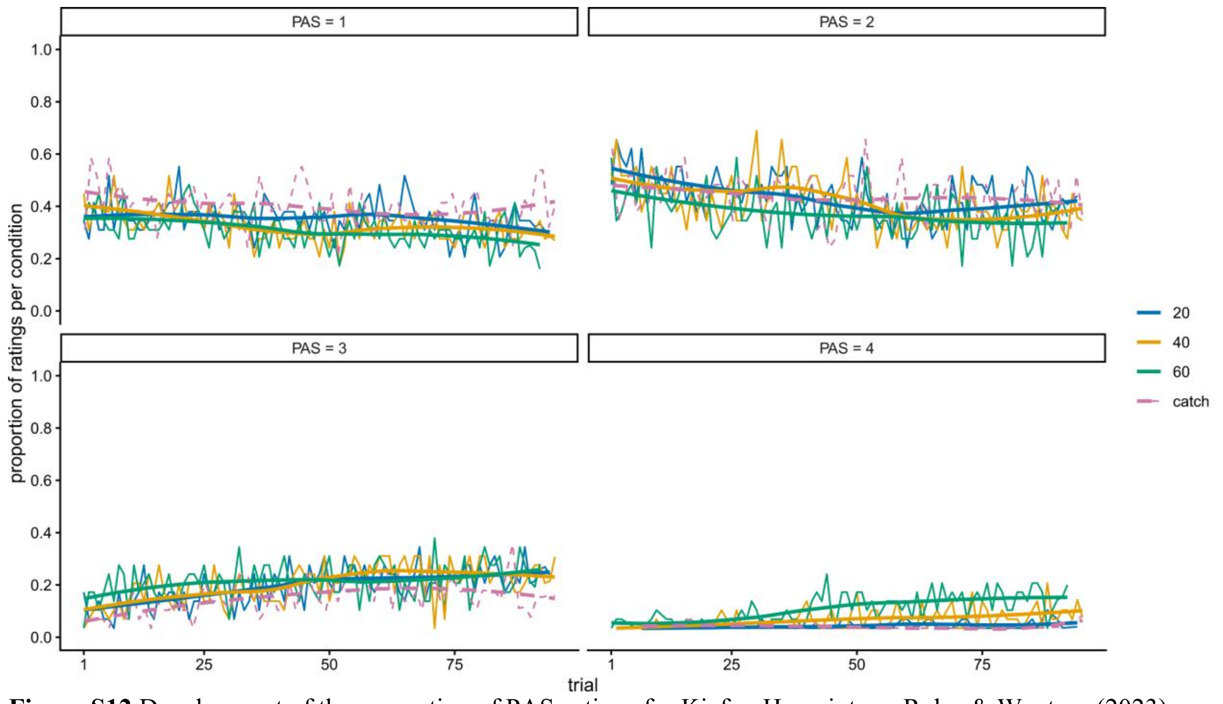

Figure S12 Development of the proportion of PAS-ratings for Kiefer, Harpaintner, Rohr, & Wentura (2023).

Supplementary references

Berger, A., Kunde, W., & Kiefer, M. (2024). Dynamics of task preparation processes revealed by effect course analysis on response times and error rates. *Scientific Reports*, *14*(1), 1–16. https://doi.org/10.1038/s41598-024-54823-1

Kiefer, M., Harpaintner, M., Rohr, M., & Wentura, D. (2023). Assessing Subjective Prime Awareness on a Trial-by-Trial Basis Interferes With Masked Semantic Priming Effects. *Journal of Experimental Psychology: Learning Memory and Cognition*, *49*(2), 269–283. https://doi.org/10.1037/XLM0001228

R Core Team. (2020). *R: A language and environment for statistical computing*.

Ramsøy, T. Z., & Overgaard, M. (2004). Introspection and subliminal perception. *Phenomenology and the Cognitive Sciences*, *3*(1), 1–23. https://doi.org/10.1023/B:PHEN.0000041900.30172.E8

Wentura, D., Rohr, M., & Kiefer, M. (2025). Does affective processing require awareness? On the use of the Perceptual Awareness Scale in response priming research. In *Journal of Experimental Psychology: General* (Vol. 154, Issue 1, pp. 128–151). American Psychological Association. https://doi.org/10.1037/xge0001648
